# The structure of the superior and inferior parietal lobes predicts inter-individual suitability for virtual reality

**DOI:** 10.1038/s41598-021-02957-x

**Published:** 2021-12-08

**Authors:** Chihiro Hosoda, Kyosuke Futami, Kenchi Hosokawa, Yuko Isogaya, Tsutomu Terada, Kazushi Maruya, Kazuo Okanoya

**Affiliations:** 1 Department of Life Science Graduate School of Arts and Sciences, The University of Tokyo, 3-8-1 Komaba, meguroku, Tokyo, 153-8902 Japan; 2 Advanced Comprehensive Research Organization, Teikyo University, Tokyo, 173-0003 Japan; 3 College of Information Science and Engineering, Ritsumeikan University, Kusatsu, Shiga 525-8577 Japan; 4 Human Information Science Laboratory, Communication Science Laboratories, Nippon Telegraph and Telephone Corporation, Atsugi, Kanagawa 243-0198 Japan; 5 Department of Psychiatry, Tohoku Medical and Pharmaceutical University Hospital, Sendai, Miyagi 983-8512 Japan; 6 Department of Engineering, Graduate School of Engineering, Kobe University, Kobe, Hyogo 657-8501 Japan

**Keywords:** Predictive markers, Neuroscience

## Abstract

The global virtual reality (VR) market is significantly expanding and being challenged with an increased demand owing to COVID-19. Unfortunately, VR is not useful for everyone due to large interindividual variability existing in VR suitability. To understand the neurobiological basis of this variability, we obtained neural structural and functional data from the participants using 3T magnetic resonance imaging. The participants completed one of two tasks (sports training or cognitive task) using VR, which differed in the time scale (months/minutes) and domain (motor learning/attention task). Behavioral results showed that some participants improved their motor skills in the real world after 1-month training in the virtual space or obtained high scores in the 3D attention task (high suitability for VR), whereas others did not (low suitability for VR). Brain structure analysis revealed that the structural properties of the superior and inferior parietal lobes contain information that can predict an individual’s suitability for VR.

## Introduction

Presently, the technology of immersive virtual reality (VR) is spreading exponentially in many fields of daily life, beyond the entertainment sector. In the new “COVID-19” era, VR will be used in an increasing number of situations. Particularly, the usefulness of VR was demonstrated in several fields as a new method for expanding or recovering human ability, such as specialized skill acquisition (e.g., surgical training^1^, sports training^2^, education for healthy people^3^, health care^4^, rehabilitation^5^, therapy for mental illness^6^, pain^7^, Parkinson’s disease^8^, and children with a developmental disability^9^).

As the fields in which VR use for expanding or recovering human ability increase, the certainty that VR use is an effective method for most people is crucial. Nevertheless, prior work reported that manipulation in the VR space did not have a practical impact on all people, namely, suitability for VR use varied significantly among individuals. There is marked interindividual variability in the suitability for the use of VR. In fact, several factors have been proposed to modulate the suitability for VR use, such as gender^10^, preference for the VR content^11^, competitive spirit^12^, anxiety, levels of self and spatial embodiment, sensorimotor rhythm desynchronization^13^, and simulator sickness symptoms^14^.

However, an essential question remained unanswered in all of those empirical observations—what is the neuronal mechanism underlying the suitability for VR? Likely, the superior parietal lobule (SPL), inferior parietal lobule (IPL), and basal ganglia underly the VR suitability. Although there is no direct evidence for their involvement in the suitability for VR, the SPL and IPL have been implicated in functions that are relevant to the suitability for VR and that require the extraction of three-dimensional (3D) shape representations for manipulating objects physically^15^. The SPL is involved in spatial tracking^16,17^ and grasping and eye movements^18^ and is key to stereopsis^18^ and depth perception^19,20^.

Other factors that support this idea include the involvement of the parietal lobe and occipital lobe in 3D visual fatigue^21^, and the association between the basal ganglia, especially the caudate nucleus (CN), and dizziness^22^ and motion sickness^23^. Moreover, the CN is critically involved in memory for visual object information, such as the encoding of spatial and response attributes (egocentric localization)^24^. Furthermore, patients with stroke involving the basal ganglia, including the CN, exhibit loss of attention and spatial neglect^25^.

The present study aimed to address the hypotheses from a brain structural perspective, for the following reasons. First, because some individuals have significantly lower suitability for VR, i.e., using long-term sports training in the virtual space, people without VR suitability cannot improve their performance in the real world, and only those with VR suitability can expand their abilities. We hypothesized that this can be attributed to differences in brain structure and function, especially in the SPL, the IPL, and the CN. To address this hypothesis, we administered long-term VR sports training and tested whether patterns in specific brain areas, particularly the SPL, IPL, and CN, predicted the ability/inability to acquire a high benefit from the VR training (high/low VR suitability).

Second, because high visuospatial attention regarding depth perception in the VR space is an essential element for improving performance in the real world^26^, we applied the multiple object tracking (MOT) task involving the frontoparietal and temporal systems^27,28^ with 2D and 3D conditions to examine individual differences in attentional functions in depth. Subsequently, to verify the generalization in another task of the predictor for VR suitability, we tested whether the predictor of VR suitability created from VR sports training could also predict the suitability for VR short-term attention tasks (MOT).

## Results

### Half of the individuals who were trained in serve–return in the virtual space exhibited improvement of the skill in the real world

**Figure 1:**
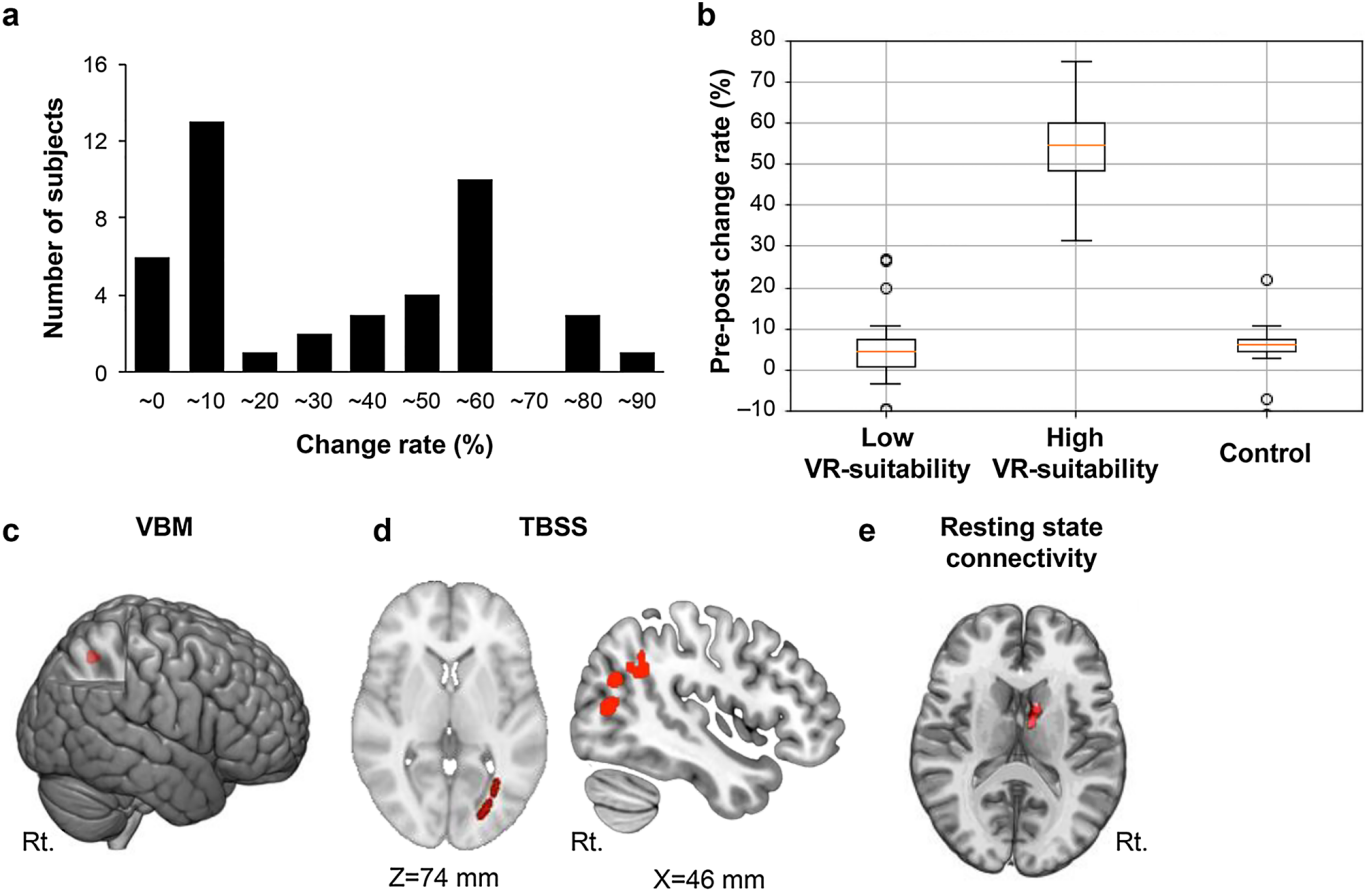
Behavioral and imaging results of serve–return training with VR. (**a**) Pre–post change rate of the return test in the actual gym after VR serve–return training. (**b**) The boxplot shows the significant difference in the change rate of pre–post VR training for the two groups, which were clustered according to the change rate. (**c**) The high VR suitability group in the serve–return training had a significantly greater GM volume in the right SPL (*P* < 0.05, FWE-corrected). (**d**) More organized fiber connectivity beneath the right IPL (right) and the occipital lobe was observed in the high VR suitability vs. the low VR suitability groups (*P* < 0.05, FWE-corrected). (**e**) The high VR suitability group in the serve–return training had a significantly greater IPL–caudate nucleus functional activity (*P* < 0.05, FWE-corrected).

To assess whether VR-based training had the effect of improving return skills in real-world badminton players, a 4 week daily VR-based serve–return training intervention was conducted. Expectedly, not all participants in VR training exhibited improvement in their serve–return abilities in the real world. The histogram of the improvement rate showed a binominal distribution (Fig. 1a), and the average pre- to post-serve–return training change rate in the actual badminton court was 31.1% (SD 27.7%) in the training group. The subjects in the control group exhibited a pre- to post-serve–return training change rate in the actual badminton court of 5.3% (SD 7.3%). The subjects who underwent VR training were clustered into two groups by applying the k-means method to the change rate. The number of clusters set in this study (k = 2) was reasonable because our purpose was to classify the existence of VR suitability and its silhouette score (more than 0.72)^29^. Subjects with a high growth rate were defined as the high VR suitability group; conversely, those with low growth rate scores were defined as the low VR suitability group. Consequently, 24 individuals had high VR suitability, and 20 had low VR suitability. The box-beard diagram depicted in Fig. 1b shows the results of these two clustered groups and control group. The mean growth rate was 2.4% in the low VR suitability group (maximum, 10.0%; minimum, − 9.7%), 51.6% in the high VR suitability group (maximum, 90%; minimum, 31.6%), and 5.3% in the control group (maximum, 10.7%; minimum, − 10.8%). Significant differences between the groups (high VR suitability, low VR suitability, and control) were detected via one-way ANOVA (F(2,55) = 114.41; *P* = 0.001). Tukey’s post hoc test revealed that the change rate of the high VR suitability group (51.6% ± 3.4%) was significantly higher than that of the low VR suitability group (2.4% ± 1.2%, *P* = 0.001) and the control group (5.3% ± 1.8%, *P* = 0.001). No significant differences in visual acuity were detected among the groups (*P* = 0.9).

### The right IPL structure predicted VR suitability in sports training

We analyzed the participants’ brain structure using T1-weighted magnetic resonance imaging (MRI), diffusion-weighted MRI, and resting-state connectivity before the training. Differences in brain structure across the whole brain were compared between the high VR suitability and the low VR suitability groups. Compared with the low VR suitability, the gray matter (GM) volume in the right SPL in high VR suitability was significantly greater (*P* < 0.05, family-wise error [FWE]-corrected) (Fig. 1c, Table 1). Additionally, a significant increase in fractional anisotropy (FA) was detected from beneath the right IPL to the occipital lobe in the high VR suitability compared to the low VR suitability groups (*P* < 0.05, FWE-corrected) (Fig. 1d, Table 2). Moreover, significantly increased functional connectivity was observed between the IPL and CN in the high VR suitability compared with the low VR suitability groups (Fig. 1e). Because the MRI scans were obtained before the VR training, these findings suggest that individual differences in the right SPL and IPL structures and in IPL-CN functional connectivity have predictive value regarding whether an individual would have suitability for VR.

**Table 1:** Differences in gray matter volume between the individuals with high VR suitability and low VR suitability for each experiment.

Anatomical location	Coordinates			Z-value	*P* corrected
	x	y	z		
**Serve–return training**					
Right superior parietal lobe	20	− 49	66	4.66	0.01
**Multiple object tracking**					
Right superior parietal lobe − 20	31	− 58	52	6.34	0.00

The coordinates (x, y, z) indicate local maxima in each brain region according to the MNI template.

**Table 2:** Differences in FA between the individuals high VR suitability and low VR suitability for each experiment.

Anatomical location	Coordinates			Z-value	*P* corrected
	x	y	z		
**Serve–return training**					
Right superior parietal lobe	20	− 49	66	4.66	0.01
Right inferior occipital lobe	37	− 67	9	5.15	0.03
**Multiple object tracking**					
Right superior parietal lobe 20	31	− 58	52	6.34	0.00
Right inferior occipital lobe	− 36	− 76	9	4.45	0.03

The coordinates (x, y, z) indicate local maxima in each brain region according to the MNI template.

Thus, we evaluated whether the brain features (the GM in the right SPL, the FA in the nearby IPL, and the IPL-CN functional connectivity) could predict the high or low VR suitability. We extracted both the GM and the FA values from the SPL and IPL, and the resting-state values from the IPL-CN, as regional features exhibiting group-wise differences. For the dataset, the explanatory variables were these three brain features, and the predictive variables were the two groups (high or low VR suitability). All prediction algorithms (random forest, support vector machine, and k-NN) could predict the VR suitability at more than 80%, with the random forest algorithm showing the highest prediction accuracy (90%, F value = 0.9; Tables 3, 4). These results indicated that the high or low suitability for VR sports training could be predicted with high probability from the structures of the IPL, SPL and from the IPL-CN functional connectivity.

**Table 3:** The accuracy rate of prediction for the VR suitability in the long-term serve–return training in each algorithm.

SVM	k-NN	Random forest
81%	81%	90%

**Table 4:** The precision, recall rate, and F-values of prediction for the VR suitability in the long-term serve–return training in each algorithm.

	SVM			k-NN			Random forest		
	P	R	F	P	R	F	P	R	F
Low	0.85	0.77	0.81	0.85	0.77	0.81	0.90	0.90	0.91
High	0.78	0.85	0.81	0.78	0.85	0.81	0.90	0.90	0.90
Ave	0.81	0.81	0.81	0.81	0.81	0.81	0.90	0.90	0.90

*P* precision, *R* recall rate, *F* F value.

Finally, analyses of the MRI data (post vs. pre) of the high VR suitability group identified training-induced increases in the GM of the supplemental motor area (SMA) and thalamus compared with the Control Group (CG) (significant time-by-group interaction). The training-induced increases in GM volume in the SMA and thalamus were correlated with those of the pre- to post-training change rate in return score (r = 0.41; *P* = 0.03; r = 0.49, *P* = 0.03). These results indicated that the neural basis of the improvement in the serve–return ability in badminton differs from that of VR suitability.

### Generalization of the VR suitability predictor to 3D MOT

The results of the VR sports training suggested that the neural correlates of the VR suitability might be the SPL, IPL, and CN, which are responsible for the stereoscopic vision and depth perception^18^. To investigate the individual differences in the information procession of a dynamic 3D scene, we used the 2D and 3D MOT tasks. In the MOT, observers are asked to track pre-specified multiple moving objects (targets) by visual attention among the other moving objects of identical appearances to the target objects. The MOT involved the frontoparietal and temporal systems subserving object recognition, attention, and working memory^27,30^. The subjects performed 10 trials for 2D and 3D tasks each, and the subjects received 1 point per trial if they answered correctly; the total score was used (perfect score, 10). Finally, we tested whether the predictor of VR suitability created from VR sports training also predicts the suitability for different VR tasks (MOT).

**Figure 2:**
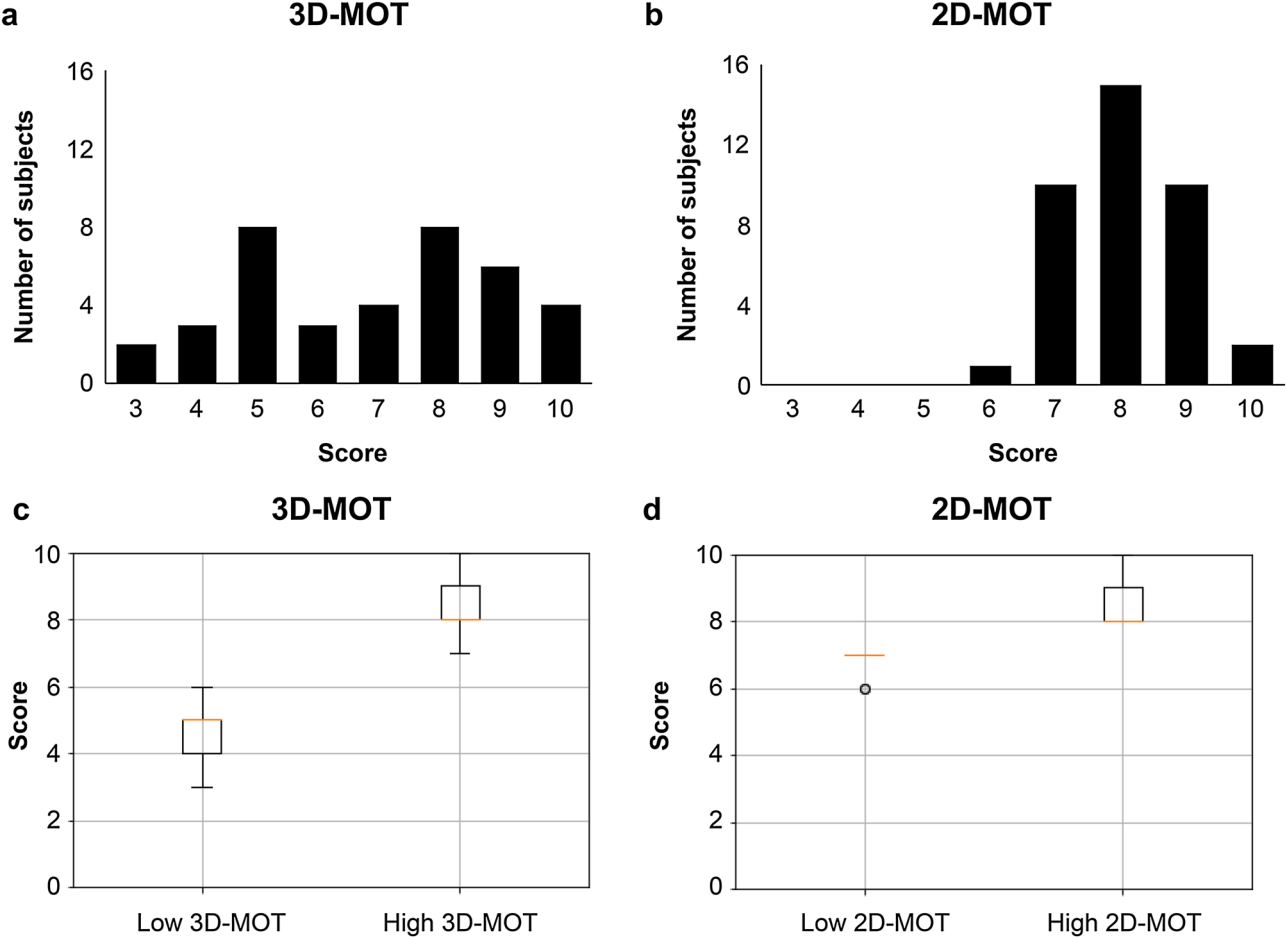
Behavioral results of 2D and 3D MOT. (**a**) The score on the 3D MOT (the number of correct answers out of 10 trials) showed a bimodal distribution. (**b**) The 2D MOT score (the number of correct answers out of 10 trials) showed a normal distribution. (**c**) The boxplot shows the significant difference in the 3D MOT score for the two groups, which were clustered according to the number of correct answers. (**d**) No significant differences were observed in the 2D MOT score for the two groups, which were clustered according to the number of correct answers.

A significant difference was detected between the 2D and 3D MOT scores (*t*-test, *P* = 0.001). On average, the score on the 2D MOT was 8.5 (SD, 1.2; maximum, 10; minimum, 8), whereas that on the 3D MOT was 6.8 (SD, 2.0; maximum, 10; minimum, 3). The distribution was bimodal in the 3D MOT (Fig. 2a), whereas it was unimodal in the 2D MOT (Fig. 2b). The subjects were clustered into two groups by applying the k-means method to the score of the 3D MOT task (silhouette score, 0.7). Consequently, 21 individuals were in the high 3D MOT group (male:female ratio, 12:9; average score, 0.84; maximum, 10; minimum, 7), and 17 were in the low 3D MOT group (male:female ratio, 10:7; average score, 4.7; maximum, 6; minimum, 3). There were significant difference in the 3D MOT score in high 3D MOT group and low 3D MOT group (*t*-test, *P* = 0.01) (Fig. 2c). No significant difference in visual acuity was detected between the two groups (*t*-test, *P* = 0.9). When the same subjects were clustered into two groups on the basis of the score on the 2D MOT task, no significant difference was detected in the score (Fig. 2d). These results suggested that the inter-individual difference in attentional function in depth might be one of the core functions of VR suitability.

Next, we evaluated whether a predictor of suitability for long VR sports training could also predict the ability for another short-term 3D or 2D attention VR task. The predictive model was created using data of all subjects in VR sports training experiments, including the explanatory variables of the brain regions (GM in the SPL, FA in the IPL, and connectivity in IPL-CN) and the predictive variables of VR suitability (high or low VR suitability) (see “Methods”). For this purpose, we evaluated whether the VR suitability predictor from the VR sports training could discriminate the high 3D MOT from the low 3D MOT groups. The SVM showed a prediction accuracy of 81% (F value, 0.8; Tables 5, 6). These results suggest that VR suitability in the two tasks with different time scales and contents might have a common neural basis. This VR suitability predictor failed to discriminate the low from the high 2D MOT.

**Table 5:** The accuracy rate of prediction for the 3D multiple object tracking from the VR suitability predictor by the sports training in each algorithm.

SVM	k-NN	Random forest
81	74	76

**Table 6:** The precision, recall rate, and F-values of prediction for the 3D multiple object tracking from the VR suitability predictor by the sports training in each algorithm.

	SVM			k-NN			Random forest		
	P	R	F	P	R	F	P	R	F
Low	0.71	0.95	0.82	0.67	0.95	0.78	0.69	0.95	0.80
High	0.93	0.64	0.76	0.92	0.55	0.69	0.93	0.59	0.72
Ave	0.83	0.79	0.79	0.80	0.74	0.73	0.81	0.77	0.76

*P* precision, *R* recall rate, *F* F value.

Finally, to test further the hypothesis that the common neural basis in the different tasks of VR suitability lies in the IPL, SPL, and CN, a reverse verification was performed. We tested whether the predictor of VR suitability from 3D MOT could predict VR suitability in sports training. The predictor of VR suitability in the 3D MOT could predict the VR suitability in sports training with 82% accuracy (SVM: F value, 0.9; Tables 7, 8). These results strongly support our hypothesis that the common neural basis in the different tasks of VR suitability lies in the IPL, SPL, and CN.

**Table 7:** Prediction accuracy of VR suitability in sports training from the VR suitability predictor by 3D-multiple object trucking in each algorithm.

SVM	KNN	Random forests
79	82	73

**Table 8:** The precision, recall rate, and F-values of VR suitability in sports training from the VR suitability predictor by 3D-multiple object trucking in each algorithm.

	SVM			KNN			Random forests		
	P	R	F	P	R	F	P	R	F
Low	0.94	0.68	0.79	1.00	0.68	0.81	0.93	0.59	0.72
High	0.68	0.94	0.79	0.70	1.00	0.82	0.62	0.94	0.75
Ave	0.83	0.79	0.79	0.87	0.82	0.81	0.80	0.74	0.73

*P* precision, *R* recall rate, *F* F value.

### Serve–return training with VR induced neuroplastic changes in subjects with high VR suitability

**Figure3:**
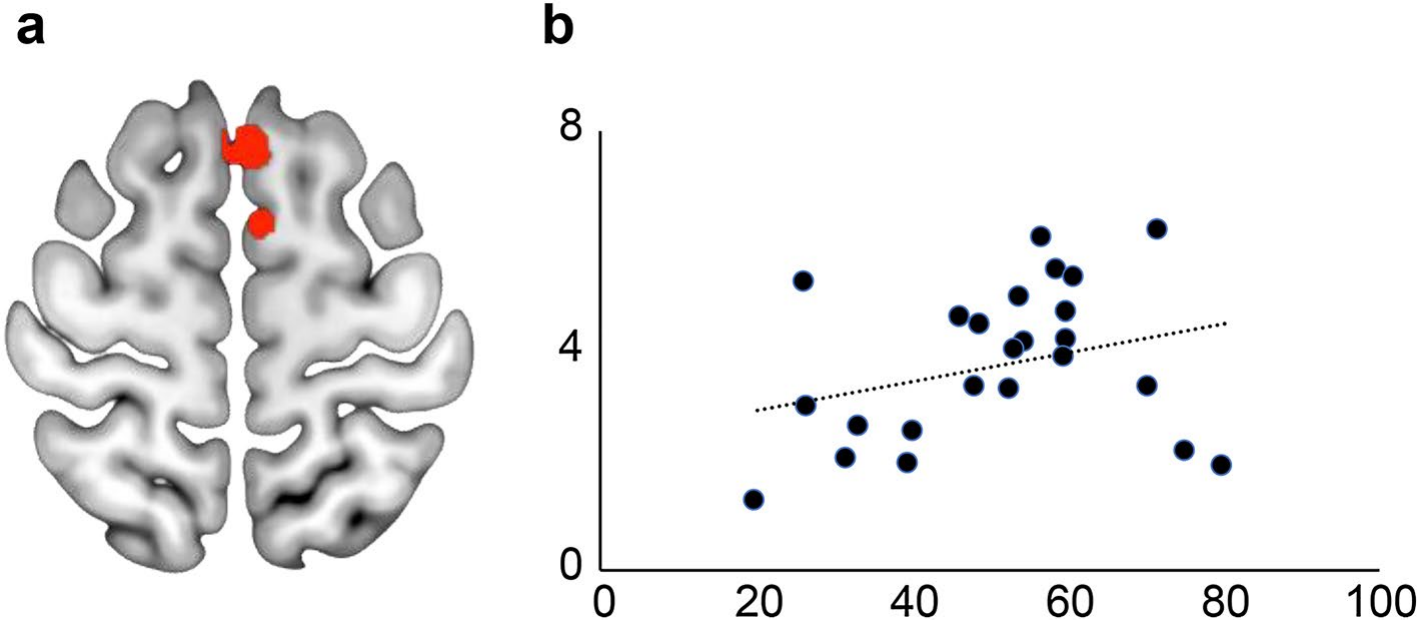
GM changes after serve–return training in the SMA within the group with high suitability for VR. (**a**) Changes in GM in the SMA before and after serve–return training in the group with high suitability for VR (**b**) A correlation was found between the change rate in serve–return score and the change rate of SMA.

To explore the training-induced changes observed in the subjects with high VR suitability in badminton serve–return training, we conducted a 2 × 2 mixed repeated-measures ANOVA using time (before and after training) as a within-subject variable and group (with VR and without VR) as a between-subject variable. These analyses identified training-induced increases in GM of the supplemental motor cortex and thalamus (Fig. 3a), and increases in FA in the cerebellum. The training-induced increase rate in GM volume in the SMA was correlated with that of the serve–return ability score (r = 0.41, *P* = 0.03) (Fig. 3b).

## Discussion

We conducted a long-term VR-based badminton serve–return training among experienced badminton players and demonstrated that only subjects with high VR suitability exhibited improvement of their skills in the real world. Moreover, we demonstrated for the first time that individual differences in VR suitability could be predicted with high probability based on the brain functions and structures of the SPL, IPL, and CN. This “VR suitability predictor” could also discriminate subjects with high performance from those with a low performance in a VR-based 3D attention task.

A growing body of evidence indicates that the SPL and IPL are consistently active and are necessary for spatial task performance^31,32^ and binocular depth^19,33^. The SPL is involved in computing target positions in the egocentric reference frame for the immediate control of reaching, grasping, and eye movements^31,32^ and is suggested to be one of the higher centers involved in stereopsis^18^. In turn, the IPL plays an important role in sustained attention^18^ and is engaged in action planning^34,35^, visuomotor control^36,37^, encoding^38^, storage^39^, and representation of action sequences^40^. These findings prompted us to explore SPL and IPL structural and functional differences between the subjects with VR suitability and those without VR suitability.

Besides localized structural differences in the GM volume in the SPL between individuals with high and low VR suitability, we detected differences in FA values extending from the occipital to the parietal lobe, including the nearby IPL. The depth perception on stereoscopy is not localized only in the SPL and IPL, as it is also associated with early occipital areas^41^. Importantly, Nishida et al. showed that stereopsis leads to activation both the occipital lobe and the SPL^41^. The visual system consisted of two main subsystems, originate in the primary visual area (V1) and project ventrally to the inferotemporal cortex (ventral stream) and dorsally to the posterior parietal cortex (dorsal stream)^16^. These streams is involved in the localization of the objects in the space and in attention. The ventral stream is engaged in the computation of the relative disparity, on the other hands, the dorsal stream is mainly engaged in elaborating the visual input to mediate the visual control of skilled actions^42^ and in the computation of stereo depth based on a computation of the binocular correlation between the images captured by the left and right eyes^43^. Moreover, another study showed that the early involvement of the visual occipital cortex in the perception of motion in depth stimuli was followed by activation within the parietal cortex, presumably associated with attention information processing^44^. Here the high VR suitability subjects showed a greater GM volume in the SPL and IPL, a higher functional connectivity from the SPL to the basal ganglia, and higher FA values along the visual pathway, suggesting that these subjects have better depth perception and perceptual–motor coordination; thus, they showed higher suitability for VR training, especially in serve–return. This hypothesis is also consistent with the classic findings that damage to the posterior parietal area, including the IPL and SPL, results in impairments in depth perception^45^.

Several roles of visual attention are common to MOT tasks and service–return training using VR, such as recording the target position in a time- or space-divisional manner, maintaining and recording information processing for the characteristics of the target, and indexing the targets^46^. In these models, attention may play a role in both adding and facilitating information processing for the target and inhibiting information processing for nontargets^47^. In this regard, ERP measurements during the MOT task have shown that attention was primarily directed to the target^48,49^. Considering these studies, in the information processing in the 3D dynamic scene, among the subjects with higher suitability for VR, the attention to the depth might be modulated to facilitate the target processing in specific contexts, which plays an important role in the VR space. Conversely, for those who had low suitability for VR, the depth of information might have become a distractor for facilitating the target processing in specific contexts.

In immersive virtual environments, the distance is underestimated^50,51^ because technological characteristics of HMD make presentation of precise depth cues problematic such as an accommodation-convergence mismatch. There were likely to be individual differences in the inaccuracy of distance judgment in VR space^50,51^.

On the other hand, human vision system weights each of the depth cues, such as binocular disparity, texture gradients, shading, motion parallax, and accommodation, and combines them to obtain a reliable 3D structure. The degree of this weighting could be varied by feedback learning^52^. It is conceivable that individual differences in visual experience (learning) accumulated on a daily basis might produce individual differences in the weighting ratios of the various cues required to construct the 3D structure. Therefore, the individual differences in VR suitability might relate to the individual difference in the misperceive of the absolute egocentric distances in current HMD systems and the cue combination in multiple depth cues (i.e. the low VR acceptability may be, at least partly, explained by the high dependency of depth cues that are hardly available in the HMD displays).

Subjects with high VR suitability had a higher FA beneath the right IPL and the occipital lobe in the present study. The vertical occipital fascia (VOF), which is involved in depth perception and motion perception^53,54^, is a pathway that connects the V3 and the V5/MT+ in the dorsal visual pathway and the V4 in the ventral visual pathway^55,56^. In the visuomotor coordination task, the VOF was related to task performance^57^. Furthermore, Howells et al. found that the frontoparietal tracts, specifically the dorsal branch of the superior longitudinal fasciculus, which is responsible for visuospatial integration and motor planning, were involved in high levels of visual–motor coordination in the right upper limb^58^. The present result is consistent with these findings regarding depth perception, motion perception, and visuomotor coordination, which might be an important factor supporting VR suitability.

In the individuals with high VR suitability, the neural plasticity consisting in the increase in the GM and FA values is correlated with the performance improvement afforded by training^59–61^. This result indicated that the IPL and SPL were not involved in task-specific returning abilities; rather, they participated in VR suitability. A previous study reported structural changes in the cerebellum after badminton training^62^. Furthermore, players of racket sports are known to perform better on visuomotor tasks, which was related to the frontal and temporal areas^63^. These results also support our hypothesis.

Although the underlying neurobiological mechanisms remain unknown, the factors proposed to explain the changes in FA include the proportion of crossing fibers, axonal permeability, and cell density or axonal/dendritic arborization^64^, whereas those for GM changes include neurogenesis, gliogenesis, synaptogenesis, and vascular changes. However, the exact biological substrates underlying the changes in GM and FA remain under investigation, and the mechanisms underlying the increase in FA values, especially in the converters, require future investigation.

Although two studies have confirmed the relationship between VR suitability and the structure of the IPL and SPL, our study was limited by the fact that we were unable to assert causality between these variables, because we did not perform a knockout experiment to prove that the disruption of IPL and SPL function increases VR suitability. Moreover, we could not test the suitability for VR use in long-term cognitive tasks (e.g., math and language learning) or motor tasks other than the serve–return task or whether the promotion of functional and structural plasticity in the IPL and SPL using techniques such as the brain–machine interface will increase VR suitability. Hence, further research is required to answer these questions. To the best of our knowledge, the present findings provide the first direct evidence of the association between high suitability for VR and the IPL and SPL. We propose that the use of the structure of the IPL and SPL as an index of VR suitability may promote the development of more efficient VR applications for education and rehabilitation purposes in the near future, to meet the growing demand for a wide range of VR applications.

## Methods

### Subjects

All participants were volunteers for this study and were selected from the university student (the subjects of the serve–returning training experiment were the member of the badminton club). After obtaining approval from the reviewing Board of University of Tokyo (approval no. 348-5) and Teikyo University School of Medicine for human studies (approval no. 0326-1), our study was conducted. Standard ethical guidelines laid down by Declaration of Helsinki-World Health Organization was followed for all methods in this study. All the participants were informed about the study well in advance and MRI safety precautions were explained to all. Before the study, a signed-in informed consent form was obtained from each participant.

In the serve–return training experiments, we enrolled forty three university students participating in a badminton club (19 females) with a mean age of 19.8 years (SD ± 3.2 years; range 18–22 years) as a learning group for the serve–returning training using VR over 4 weeks. To examine the effect of skill improvement, skilled players were selected as subjects, rather than beginners. The average number of years of experience was 5.4 (SD ± 6.2; range 3–11). Fifteen subjects also in a badminton club (seven females) with a mean age of 19.5 years (SD ± 0.9 years; range 19–21 years) participated in the study as a control group.

In the multiple objects tracking experiments, we enrolled thirty eight university students (21 males; average age, 20.0 years; SD ± 0.9 years; range 18–21 years) to perform the 2D and 3D MOT using VR. All subjects were chosen through an interview and were highly motivated university students who were healthy and neurologically intact and had no history of neuropsychiatric disorders, psychotropic medication use, or head injury.

The subjects were rigorously trained to maintain fixation throughout the experiment and performed about five trials of the 2D and 3D MOT tasks, to ensure correct understanding and performance of the task. The order of the 2D and 3D tasks was randomized for each subject. We performed 10 trials for each task, and the subjects received 1 point per trial if they answered correctly; the total score was used (perfect score, 10).

### Experimental design

Before the tasks (serve–return training or 2D and 3D MOT task), all participants underwent MRI scanning (T1-weighted imaging and diffusion-weighted imaging [DWI]) using a 3T MRI scanner (Siemens PRISMA, Erlangen, Germany). Participants in the serve–return training also underwent MRI scanning after training.

### Behavioral data acquisition

#### Serve–return training

To assess the effects of sports training using VR, previous studies applied a training schedule spanning days or weeks^2,65^. In the present study, the subjects underwent a 4 week serve–return training using VR (HTC VIVE PRO; visual angle, 110°; refresh rate, 90 Hz; resolution, 2880 × 1600 pixels) with racket (HTC racket handle). On each day of the training period, the participants were required to return 80 serves to the edge of the opponent’s court in the VR space. The subject obtained 10 points if the shuttle fell within 10 cm from the edge of the service line, and 5 points if the shuttle fell within other areas on the court. Before and after VR training, to determine if the ability of serve–returning was improved, the subjects were required to undergo a similar rule of serve–return test in the real-world gym. For each of eight serves, the subject returned the serves and got a score according to the position where the shuttle fell. The total score of the 10 times of serve–return was used as the ability and compared it before and after training.

The training environment in VR was set by Unity 2018.2.0f2, and the initial speed of serve was randomly set from 30 to 100 km/h. Two types of orbit calculation, the drive shot and the clear shot, were prepared for the serve. In the drive shot, the effect of gravity was ignored, whereas the initial velocity was applied toward the target point, and a constant velocity linear motion was adopted. In the case of the clear shot, the coordinates of the midpoint between the target point and the start point were calculated, and the highest reached point was calculated from the initial velocity (h = v0 × v0/2*g*). The Z coordinate (height direction) of the midpoint was set to the highest point, and the initial velocity was given toward this point. The weight of the shuttle was set to 4*g* and was a parabolic motion affected by gravity.

#### MOT

All participants underwent the 2D and 3D versions of the MOT task using VR (HCP VIVE PRO), with 10 repetitions each. Subjects were allowed five practice runs each before performing the main trial, to understand the rules and stabilize the score.

The order of execution of the 2D and 3D tasks was randomly selected and counterbalanced. For both tasks, the percentage of correct responses was calculated as the number of correct responses after 10 trials. The subjects were allowed five practice runs each before performing the main trial.

In the 2D MOT, the stimulus was presented in a gray square area (21 × 21 cm) in the VR space. The task proceeded with the following steps: (1) a white cross with a length of 1 cm and a width of 0.1 cm was presented in the center of the square for 3 s. (2) Eight white circles with a diameter of 1.0 cm were presented anywhere in the stimulus-presentation area (randomized for each trial) for 1.5 s. At this time, the circles were presented at a location where the centers of the circles were at least 2 cm apart from each other so that the circles did not overlap with each other. (3) Four of the eight white circles (randomly selected for each trial) turned green for 4 s. (4) After returning to white from the green, all circles moved in the stimulus-presentation area in a random direction at a speed of 6 cm/s for 20 s, before stopping. The circles moved in a straight line, and the direction in which they started moving was random. The circle bounced back when it hit the wall of the stimulus-presentation area. The circles bounced off each other when the centers of the circles approached each other up to 2 cm so that the circles did not overlap with each other. (5) After the white circles stopped moving, the four circles turned green, and the numbers 1–4 were presented in the green circles in order, from left to right, for 3 s. Three of the four circles that changed to green were the first ones to turn green (three out of the four circles were randomly selected for each trial), and the remaining one was the first one to turn white (one out of the four circles was randomly selected for each trial). Subjects were asked to press a button to indicate the number of the circle that was initially white but turned green at the end. (6) The correctness of the answer and the reaction time (ms; from the presentation of the number to the answer) was recorded. The response was accepted only for 3 s after the green circle and number were presented, after which it was invalid. (7) After answering, a correct answer (the circle that was initially white but turned green after it stopped moving) was changed to red for 2 s to provide feedback to the subject. (8) All circles were turned off and only the gray stimulus-presentation area was presented for 20 s. Steps 1–8 were repeated 10 times.

In the 3D MOT, the stimulus was presented in a gray cubic area (21 × 21 × 21 cm) in the VR space. The flow of the 3D MOT task was basically the same as that of the 2D MOT. However, the stimulus was not a circle; rather, it was a sphere of 1 cm that was presented in the center of the spatial area. The moving direction of the sphere was random, including depth. The procedures used were the same as those described for the 2D MOT.

### Image data acquisition

The MRI data used in this research are obtained from 3T MRI scanner with a 64-channel phased-array receiver coil (Siemens PRISMA, Erlangen, Germany). High-resolution, three-dimensional (3D), T1-weighted anatomical images were obtained with a magnetization-prepared rapid gradient echo sequence, which was designed as follows: echo time (TE) = 2.98 ms, repetition time (TR) = 1900 ms, inversion time = 990 ms, field of view (FOV) = 192 × 176 mm, matrix size = 192 × 176, flip angle = 80°, and 1 mm^3^ isotropic voxels. We also acquired whole-brain DWI as follows: TE = 62 ms, TR = 6500 ms, matrix size = 128 × 128, FOV = 276 × 276 mm, flip angle = 90°, 68 slices, 1 × 1 × 1 mm^3^ isotropic voxels. Field map images were acquired in the same scanning space as that of the DWI (TE1 = 5.19 ms; TE2 = 7.65 ms).

We also acquired the multiband resting state, as follows: TE = 30 ms; TR = 1500 ms; slice thickness = 2 mm; voxel size = 2 × 2 mm; 180 volumes; 80 slices; and flip angle = 90°. For the resting‐state functional MRI, participants were asked to see the fixation points.

### Quantification and statistical analysis

#### Image data analysis

##### GM-VBM

T1-weighted images were subjected to voxel-based morphometry (VBM) analysis using the CAT toolbox (http://dbm.neuro.uni-jena.de/cat.html) implemented in SPM12 (http://www.fil.ion.ucl.ac.uk/spm). We implemented an optimized VBM protocol for segmentation and normalization processes, using the DARTEL (Diffeomorphic Anatomical Registration using Exponentiated Lie algebra) toolbox^66^ in SPM. The Montreal Neurological Institute (MNI) 152 standard brain template in CAT12 was adopted to do normalization of the standard space. We calculated the total intracranial volume (TIV) for all scans. The extracted GM were smoothed using a 12 mm FWHM kernel. A 0.1 absolute masking threshold were applied to the VBM data.

For the serve–return and MOT experiments, we tested the hypothesis that particular brain regions were reserved for suitability for VR. If this were the case, participants with particularly developed structures in the brain would show suitability for VR. To test this hypothesis, we performed a two-sample *t*-test using MRI data from the pretask condition in individuals with high and low suitability for VR in both the VR serve–return training experiment and MOT experiment. We performed a GLM analysis incorporating sex, age, TIV, and years of badminton experience for the serve–return training experiment, and the severe stimulation sickness score as covariates, to remove their confounding effects (*P* < 0.05, FWE-corrected), for the serve–return training experiment and MOT experiment. In the serve–return training experiment, to identify the GM changes induced by VR serve–return training, we conducted a 2 × 2 mixed repeated-measures analysis of variance using time (Pre and Post) as a within-subject variable and group (training group and control group) as a between-subjects variable.

##### DWI-TBSS

Preprocessing and analysis of DWI data were conducted with the Oxford Center for Functional MRI of the Brain (FMRIB) software library (FSL 6.0.1; http://www.fmrib.ox.ac.uk/fsl/). Pre-processing consisted in eddy-current correction, skull-stripping with the Brain Extraction Tool (BET), estimation of the diffusion tensor model at each voxel using the DTI fit tool, generating FA. After the calculation of the FA map for each participant, we implemented a voxel-wise statistical analysis of the FA data^67^ using TBSS.

Similar to the VBM analysis, we first tested whether the WM structure before each task (serve–return training and 3D MOT) could predict whether participants would have suitability for VR. To identify learning-induced reorganization of the WM, we conducted a 2 × 2 mixed repeated-measures ANOVA using time as the within-subject variable and group as the between-subject variable (*P* < 0.05, FWE-corrected), yielding FA changes specific to the VR training program.

##### Resting-state connectivity_CONN

Spatially preprocessed resting‐state functional data were analyzed using the Functional Connectivity Toolbox (CONN)^68^ running in MATLAB. CONN implements a component‐based noise correction method to reduce physiological and extraneous noise, thus providing interpretative information on correlated and anticorrelated functional brain networks^69^. Data were band-pass filtered (0.008–0.09 Hz) to reduce low‐frequency drift and noise effects^70^. The seeds are provided in the CONN software and represent the core and reproducibly demonstrated topological nodes within each resting-state network^70^. We investigated the functional networks generated from seed (IPL). To give maps of voxel‐wise functional connectivity for each seed ROI for each subject, the resulting coefficients were converted to normally distributed scores using Fisher’s transformation^70^. The value of each voxel throughout the whole brain represents the relative degree of functional connectivity with each seed^70^.

Similar to the VBM and TBSS analyses, we tested whether the resting-state connectivity before each task (serve–return training and 3D MOT) could predict whether participants would have suitability for VR. We performed a voxel‐wise statistical analysis over the entire brain using a corrected level (*P* < 0.05) before a false discovery rate correction was applied at the cluster level (*P* < 0.05).

### Predictor of suitability for the VR serve–return training

We evaluated whether the brain features (the GM in the right SPL, FA in the nearby IPL, and IPL–CN functional connectivity) could predict the level of VR suitability. We set up a 5 mm spherical VOI at the peak voxel in the right SPL for the GM, in the IPL for the FA, and in the right CN for the resting-state activity. Next, we extracted both the GM and the FA values from the SPL and IPL, and the resting-state values from the IPL-CN as regional features exhibiting group-wise differences. For the dataset, the explanatory variables were these three brain features, and the predictive variables were the two groups (high or low VR suitability). A machine learning algorithm was applied to the dataset, and a tenfold cross-validation was performed. To validate the appropriate prediction algorithms, we used different prediction algorithms, such as random forest, SVM, and k-NN. Parameters of the classifier were set as follows. Parameters of the classifier parameters were set as follows so as to make the training model high accuracy. For SVM, rbf was used as the kernel. For k-NN, the number of neighbor objects was tuned from between 1 and 5. For random forest, gini coefficients were used as a criterion for splitting. Weka (Waikato Environment for Knowledge Analysis) was used for a software for machine learning. We calculated the precision, recall, F-values for the training group in the serve–return task and in 3D MOT.

### Generalization of the predictor of VR suitability

To test the generalization of the predictor of VR suitability, we evaluated whether the predictor of VR suitability obtained from VR sports training could discriminate the high 3D MOT group from the low 3D MOT group. Firstly, we created the predictive model using data (the GM and the FA values from the SPL and IPL, and the resting-state values from the CN) from all subjects in the sports training.

Next, from the subjects who performed MOT, we extracted the GM and the FA values from the SPL and IPL, and the resting-state values from the CN that exhibited the same coordinate of the regional features exhibiting group-wise differences in the VR sports training experiments. Subsequently, to test whether the VR suitability predictor obtained from the VR sports training could discriminate the high 3D MOT from the low 3D MOT groups, we applied the regional features from the subjects in 3D MOT to the predictor from the sports training. To validate the appropriate algorithms, we used four different algorithms, i.e., random forest, SVM, and k-NN.

We also tested whether the VR suitability in 3D MOT (high 3D MOT or low 3D MOT) could be predicted by the predictor of VR suitability created by the 3D MOT. For the dataset, the explanatory variables were these three brain features, and the predictive variables were the two groups (high or low 3D MOT).

Finally, a reverse verification was performed to test further the hypothesis that the common neural basis in the different tasks of VR suitability lies in the IPL, SPL, and CN. We tested whether the predictor of VR suitability from 3D MOT could predict VR suitability in sports training. The predictive model was created using data from all subjects in the sports training experiments.

## Data Availability

The data supporting the findings of this study is available upon reasonable request to the corresponding author (for verification purposes only and not for future studies). Concerning the raw brain imaging data, two participants did not agree to share, and thus data of these two participants is not available.
